# Gynecologic oncologists’ attitudes and practices relating to integrative medicine: results of a nationwide AGO survey

**DOI:** 10.1007/s00404-017-4420-y

**Published:** 2017-06-08

**Authors:** Evelyn Klein, Matthias W. Beckmann, Werner Bader, Cosima Brucker, Gustav Dobos, Dorothea Fischer, Volker Hanf, Annette Hasenburg, Sebastian M. Jud, Matthias Kalder, Marion Kiechle, Sherko Kümmel, Andreas Müller, Myrjam-Alice T. Müller, Daniela Paepke, Andre-Robert Rotmann, Florian Schütz, Anton Scharl, Petra Voiss, Markus Wallwiener, Claudia Witt, Carolin C. Hack

**Affiliations:** 1 Klinik und Poliklinik für Frauenheilkunde, Technische Universität München, Munich, Germany; 2 Frauenklinik des Universitätsklinikums Erlangen, Friedrich-Alexander-Universität Erlangen-Nürnberg, Comprehensive Cancer Center Erlangen-Europäische Metropolregion Nürnberg (CCC ER-EMN), Erlangen, Germany; 3 Zentrum für Frauenheilkunde, Klinikum Bielefeld Mitte, Bielefeld, Germany; 4 Universitätsklinik für Frauenheilkunde und Geburtshilfe, Paracelsus Medizinische Privatuniversität, Nuremberg, Germany; 5 Klinik für Naturheilkunde und Integrative Medizin der Kliniken Essen-Mitte, Essen, Germany; 6 Klinik für Gynäkologie und Geburtshilfe, Klinikum Ernst von Bergmann, Potsdam, Germany; 7 Frauenklinik und Brustzentrum Nathanstift, Klinikum Fürth, Fürth, Germany; 8 Klinik und Poliklinik für Geburtshilfe und Frauengesundheit, Universitätsmedizin Mainz, Mainz, Germany; 9 Klinik für Frauenheilkunde und Geburtshilfe, Philipps-Universität Marburg, Marburg, Germany; 10 Interdisziplinäres Brustkrebszentrum der Kliniken Essen-Mitte, Essen, Germany; 11 Frauenklinik, Städtisches Klinikum Karlsruhe gGmbH, Karlsruhe, Germany; 12 Frauenarztpraxis am Hochdahler Markt, Erkrath, Germany; 13 Praxis für Frauenheilkunde, Geburtshilfe und Naturheilkunde, Rodgau, Germany; 14 Universitätsfrauenklinik, Universitätsklinikum Heidelberg, Heidelberg, Germany; 15 Brustzentrum Klinikum St. Marien Amberg, Amberg, Germany; 16 Institut für komplementäre und integrative Medizin, UniversitätSpital Zürich und Universität Zürich, Zurich, Switzerland; 17 Department of Gynecology and Obstetrics, University Hospital Erlangen, Friedrich-Alexander University Erlangen-Nuremberg, Comprehensive Cancer Center Erlangen-European Metropolitan Area Nuremberg (CCC ER-EMN), Universitätsstrasse 21-23, 91054 Erlangen, Germany

**Keywords:** Integrative medicine, Complementary medicine, Gynecologic oncology, Breast cancer, Oncologists’ attitudes, Survey

## Abstract

**Purpose:**

The growing popularity and acceptance of integrative medicine is evident both among patients and among the oncologists treating them. As little data are available regarding health-care professionals’ knowledge, attitudes, and practices relating to the topic, a nationwide online survey was designed.

**Methods:**

Over a period of 11 weeks (from July 15 to September 30, 2014) a self-administered, 17-item online survey was sent to all 676 members of the Research Group on Gynecological Oncology (Arbeitsgemeinschaft Gynäkologische Onkologie) in the German Cancer Society. The questionnaire items addressed the use of integrative therapy methods, fields of indications for them, advice services provided, level of specific qualifications, and other topics.

**Results:**

Of the 104 respondents (15.4%) using integrative medicine, 93% reported that integrative therapy was offered to breast cancer patients. The second most frequent type of tumor in connection with which integrative therapy methods were recommended was ovarian cancer, at 80% of the participants using integrative medicine. Exercise, nutritional therapy, dietary supplements, herbal medicines, and acupuncture were the methods the patients were most commonly advised to use.

**Conclusion:**

There is considerable interest in integrative medicine among gynecological oncologists, but integrative therapy approaches are at present poorly implemented in routine clinical work. Furthermore there is a lack of specific training. Whether future efforts should focus on extending counseling services on integrative medicine approaches in gynecologic oncology or not, have to be discussed. Evidence-based training on integrative medicine should be implemented in order to safely guide patients in their wish to do something by themselves.

## Introduction

Complementary and integrative medicine is becoming increasingly popular with gynecological patients. At present, 38–60% of all cancer patients in Western industrialized countries take advantage of complementary and alternative medicine (CAM) during the course of their disease and to support their treatment [1]. In the case of breast cancer, the figure is even as high as 90% [2, 3]. Breast cancer patients and gynecological cancer patients in particular, are the group with the highest percentage usage of integrative methods [1, 4, 5]. Women’s willingness to take the initiative in relation to these treatments is generally greater than men’s, and women are highly motivated in relation to their disease, with good compliance and considerable perseverance.

The Academic Consortium for Integrative Medicine and Health in the USA has described integrative medicine as follows: “Integrative medicine and health reaffirms the importance of the relationship between practitioner and patient, focuses on the whole person, is informed by evidence, and makes use of all appropriate therapeutic and lifestyle approaches, healthcare professionals and disciplines to achieve optimal health and healing” [6]. In this approach, complementary methods that aim to contribute to holistic care are integrated into present-day medical practices. The complementary procedures—for the most part based on experience—are to be regarded as a supplement to the current scientific, evidence-based medical system, not as a substitute for it. We classify the integrative medicine into five sub-groups: whole medical systems (e.g., homeopathy, naturopathic treatments, Ayurveda), mind/body-based interventions (e.g., meditation, chi gong, yoga), body-based therapies (e.g., massages, sports, chiropractic), biological-based therapies (e.g., phytotherapy, vitamins, enzymes) and energy-based methods (e.g., electrotherapy, hyperthermia, ultrasound therapy). Common methods in integrative medicine include homeopathy, anthroposophic medicine, in particular mistletoe therapy, classic naturopathic treatment, phytotherapy, traditional Chinese medicine (TCM) including acupuncture, sports, nutritional approaches, vitamin products, mineral nutrients, dietary supplements and relaxation therapies [7].

Previous studies on CAM have mainly addressed the frequency and methods involved in the complementary therapies used, as well as the patients’ motivation, objectives, information sources, and characteristics [8–12]. Most of the breast cancer patients show high interest in CAM [13, 14]. However, little is known about the acceptance and use of integrative medicine by gynecological oncologists in Germany. At the moment the overall qualified access to counseling on integrative medicine is not available. There is a lack of data with regard to the provision of information, competences, qualifications, and structures. Little is also known about the concrete ways in which integrative medical therapies are implemented and used in the field of gynecology.

The present study was therefore carried out to evaluate and examine the degree of acceptance, usage, and implementation of integrative medicine among gynecological oncologists in Germany.

## Materials and methods

A self-administered 17-item online survey was sent between July 15, 2014 and September 30, 2014 to all 676 members of the Research Group on Gynecological Oncology of the German Cancer Society (Arbeitsgemeinschaft Gynäkologische Onkologie, AGO).

The survey was developed and distributed by the Research Group on Integrative Medicine (AG IMed), which was founded on June 28, 2013. This group of gynecological oncologists focuses on the clinical, scientific, and organizational aspects of integrative medicine in oncology. It supports scientific research and cooperation in the field of integrative medicine and also encourages the implementation of approved integrative therapy approaches and regular consultation hours for the purpose, in order to integrate these into standard oncologic care.

For the validation, the questionnaire of the survey was tested in the AG IMed Research Group on Integrative Medicine, consisting of 20 members, and evaluated using a specially developed assessment sheet. After the validation, the questionnaire was modified with a view to improving comprehension, functionality and expenditure of time.

The online survey was sent to all 676 members of the AGO via e-mail. Participation was voluntary and anonymous. The first e-mail and call for participation was launched in July 2014, and a reminder e-mail was sent in September to members who had not yet responded, until the original deadline had passed.

The survey contained 17 questions including demographic data and items on the use of integrative therapy methods, fields of indications, consulting services, level of specific qualifications, and other topics. There are no standardized questionnaires for professionals on this topic. So we had to develop our own questionnaire for this survey. The time required to respond to it was approximately 10 min.

Data were analyzed using IBM SPSS Statistics, version 24 (IBM Corp., Armonk, New York, USA). Statistical evaluation consisted of descriptive analysis. Total amounts and percentages were calculated.

## Results

In all, 104 of the 676 AGO members participated in the survey (32.7% women, 67.3% men). This represents a response rate of 15.4%. The respondents’ median age was 47 years (range 30–71 years). The majority (56.7%) had medical degrees (M.D./Dr. med.), and 35.6% of the responding physicians also had higher qualifications, such as associate professor or professor. Seventy-six percent of the participating members were working at certified breast cancer centers and 56.7% of them at certified gynecological oncology centers. This is consistent with the demographic structure of the AGO. The percentage of men with 66% is almost twice as high as that of women’s with 34%. 54.1% of the AGO members bear the title M.D., 29.3% professor and 9.2% associate professor. There are 77.4% full members and 19% associated members. The age ranges from 26 to 86 years with a mean age of 51 years.

**Fig. 1:**
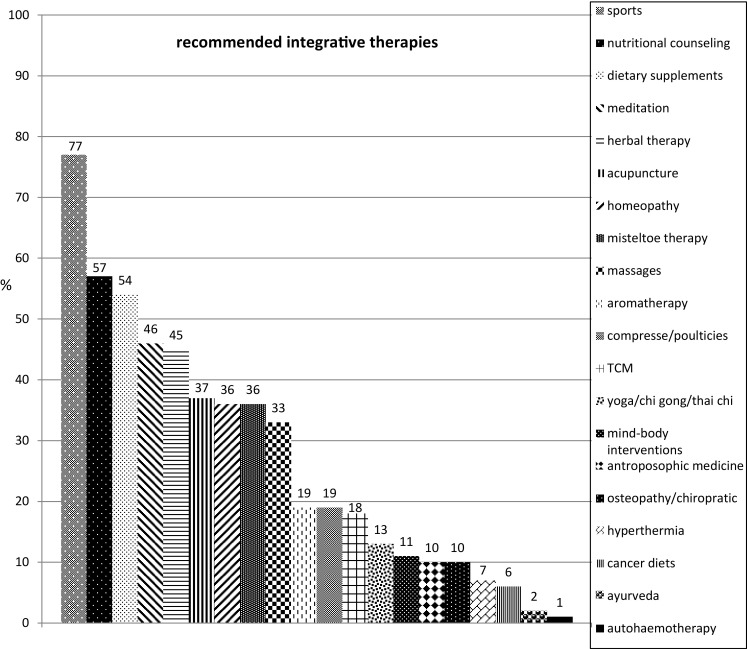
Frequencies of recommended integrative therapy methods. *TCM* traditional Chinese medicine; *n* = 83 (*n* = 21 not applicable); multiple responses were allowed

**Fig. 2:**
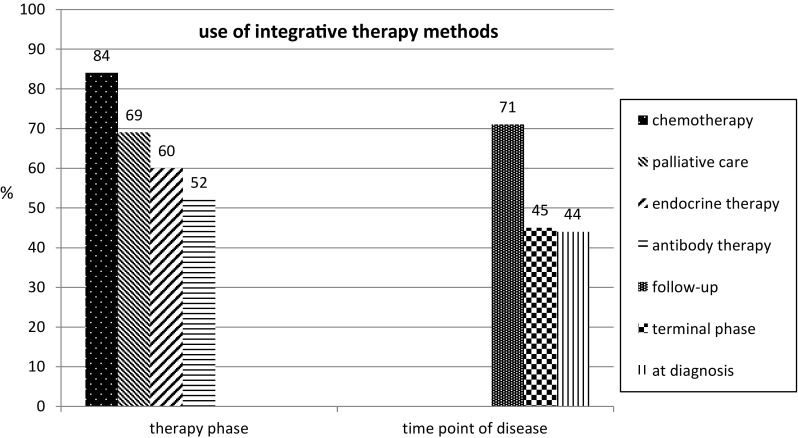
Frequency with which advice about integrative therapy methods is given during specific phases of treatment and at different time points in the disease; there are no differences in the kind of methods at the different phases and time points of use; *n* = 86 (*n* = 18 not applicable); multiple responses were allowed

**Fig. 3:**
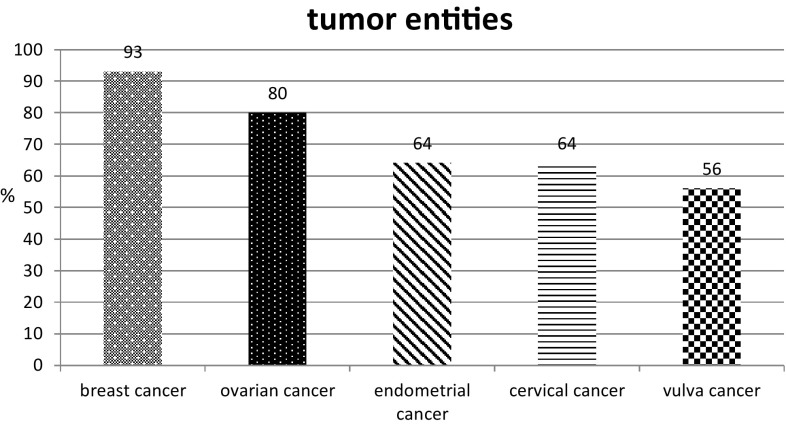
Tumor entities in connection with which integrative therapies were suggested. The most common gynecological carcinomas are represented; *n* = 87 (*n* = 17 not applicable); multiple responses were allowed

The form of integrative therapy method most commonly recommended amongst the participants was regular physical exercise, followed by nutritional counseling and advice on dietary supplements (Fig. 1). The main indications were fatigue, nausea, depression, menopausal symptoms, and sleeping disorders (Table 1). When they were asked at which point of time they provided advice about integrative therapy, most of the oncologists indicated that it was during follow-up care for their patients. With regard to the treatment phase, counseling on integrative medicine was most frequently provided during chemotherapy (Fig. 2). Most of the physicians surveyed and using integrative medicine (93%) reported that they used integrative therapy methods with breast cancer patients. The second largest patient group to whom integrative therapies were suggested consisted of ovarian cancer patients, at 80% of the participants using integrative medicine (Fig. 3). This is not in contrast to their statement that two-thirds stated that integrative medicine is not routinely implemented in the therapy concept, because implementation in the routine is still more than a recommendation from time to time, for example consultations of integrative medicine or professional counseling on CAM.

**Table 1:** The main indications for using integrative therapies; *n* = 82 (*n* = 22 not applicable); multiple responses were allowed

Indication	*n*	%
Fatigue	65	79.3
Nausea and vomiting	61	74.4
Depression	59	72.0
Menopausal symptoms	59	72.0
Sleeping disorders	59	72.0
Loss of appetite	58	70.7
Joint pain	44	53.7
Polyneuropathy	43	52.4
Abdominal discomfort	42	51.2
Cognitive impairments	39	47.6
Mucositis	35	42.7
Hand–foot syndrome	34	41.5
Pain	34	41.5
Radiation-induced dermatitis	30	36.6

Counseling on applicable integrative therapies is mainly provided by the physicians themselves (93%), and secondly by dietitians, collaborating partners (e.g., specialized centers, nonmedical practitioners, etc.), and breast care nurses (Table 2). The counseling was performed according to the differences in the professional qualification. The additional qualifications most often held by those providing advice were in naturopathy (48.6%), followed by nutritional medicine (30.5%) and acupuncture (29.2%). All of these are qualifications that are officially recognized by the relevant regional medical councils. Other areas in which qualifications were held were homeopathy, traditional Chinese medicine (TCM), phytotherapy, etc. Table 3 lists the proportions of advisers holding the different types of qualifications.

**Table 2:** Professions frequently providing counseling about relevant integrative therapies

Profession	*n*	%
Physician	80	92.0
Nutritionist	39	44.8
Collaborating partners	39	44.8
Breast care nurse	36	41.4
Sports researcher	17	19.5
Study nurse	10	11.5
Mind–body therapist	5	5.7

Collaborating partners were, for example, clinical centers offering integrative medicine, professionals for CAM; *n* = 87 (*n* = 17 not applicable); multiple responses were allowed

**Table 3:** Additional qualifications for therapy (registered with regional medical councils) of individuals providing advice about integrative medicine

	*n*	%
Naturopathy	35	48.6
Acupuncture	21	29.2
Nutritional counseling	21	29.2
Homeopathy	17	23.6
Manual therapy/chiropractic	6	8.3
Further additional qualifications		
Traditional Chinese medicine	16	22.2
Herbal therapy	11	15.3
Anthroposophic medicine	10	13.9
Neural therapy	7	9.7

Naturopathy, acupuncture, nutritional counseling, homeopathy and manual therapy/chiropractic are recognized qualifications for physicians in Germany; *n* = 72 (*n* = 32 not applicable); multiple responses were allowed

Two-thirds of the gynecologic oncologists surveyed stated that integrative therapy concepts were not implemented in routine clinical work, but 64.7% of them indicated that they were planning to do so. The main reasons given in the further comments section for not implementing integrative medicine in their hospitals were staff shortages, a lack of specific knowledge and qualifications, as well as a lack of scientific evidence on the efficacy of the therapies concerned. Around half of the physicians (55.5%) stated that integrative therapy methods are not reimbursed and are therefore not profitable for the hospitals.

## Discussion

Patients with breast cancer and gynecological cancer are known to be one of the patient groups who make use of integrative medicine most often [15]. These patients wish to receive advice from their oncologists not only about conventional medicine, but also about complementary therapy methods during and after the disease [13, 16]. More and more oncologists in Germany are beginning to appreciate this need and the importance of offering professional advice regarding integrative medicine.

As the survey shows, however, there is still a lack of widespread implementation of integrative therapy approaches in routine clinical work. Only one-third of the oncologists who responded had routinely offered complementary counseling to their patients. The main reasons for this are a lack of knowledge and professional training. This finding is consistent with the report by Muecke et al., who showed that education and training are the most essential requirements for physicians to enable them to implement CAM methods [17]. Other national and international studies have also confirmed the finding that there is generally a high level of interest in integrative therapy approaches amongst physicians, but that specific training in CAM methods is lacking [17–22]. Comparison with existing data is difficult, as the studies are not mainly aimed at oncologists, but rather at physicians in general, so that the groups of patients involved differ. Conrad et al. conducted a large survey on professionals in palliative care regarding attitudes toward CAM. Acceptance of CAM was high (92% for complementary medicine). Only 21% think themselves adequately informed [23]. Another study evaluated the attitude of employees of a university clinic to complementary and alternative medicine in oncology is also in line with our results. Most participants were interested in complementary medicine and a substantial part would use CAM. But they were not trained on this topic [24]. In addition, most published data on complementary therapy methods have concentrated on the patient’s point of view, mainly examining their acceptance and use of integrative medicine.

With regard to the patient groups identified in the present survey, the largest group to whom the oncologists recommended complementary therapies was breast cancer patients—a finding that is consistent with earlier studies [5, 25–27]. This again underlines the importance and need for gynecologic oncologists to acquire knowledge about verified integrative therapy approaches in order to offer counseling in this field.

The findings should be evaluated in light of the limitations of the study. The national survey was only addressed to AGO members and not to other hospitals or oncologists who also counsel breast cancer and gynecological cancer patients in Germany. In addition, there might have been some overlap among the responses if several AGO members from a single hospital were responding. Finally, the response rate and thus the final number of participants (*n* = 104) were quite low.

In view of the results of this survey and the existing data, future efforts should focus firstly on improving acceptance, and secondly on extending counseling services for integrative medicine approaches in gynecologic oncology. To ensure this, the existing additional training courses organized by the various regional medical councils and specialized research groups should be taken advantage of more frequently. The aim should be to provide more gynecologic oncologists in hospitals and oncologists’ offices with sound expertise, to enable them to provide guidance for patients. Particularly during the last few years, there has been increasing research and there is consequently growing evidence for integrative medicine in oncology. Although further research is still needed, oncologists can provide their patients with evidence-based treatment approaches and recommendations.

Evidence-based training on integrative medicine should be implemented in order to safely guide patients in their wish to do something by themselves. The educated attending physicians could better raise the topic of integrative medicine and encourage evidence-based complementary treatments for ensuring individualized, holistic, and patient-centered care.
